# The nature of the human T cell response to the cancer antigen 5T4 is determined by the balance of regulatory and inflammatory T cells of the same antigen-specificity: implications for vaccine design

**DOI:** 10.1007/s00262-018-2266-1

**Published:** 2018-11-07

**Authors:** Matthieu Besneux, Alexander Greenshields-Watson, Martin J. Scurr, Bruce J. MacLachlan, Adam Christian, Michael M. Davies, Rachel Hargest, Simon Phillips, Andrew Godkin, Awen Gallimore

**Affiliations:** 1 Division of Infection and Immunity, Henry Wellcome Building, Cardiff University, Health Park, Cardiff, CF14 4XN UK; 2 Department of Colorectal Surgery, University Hospital of Wales, Cardiff, UK; 3 Department of Pathology, University Hospital of Wales, Cardiff, UK; 4 Department of Gastroenterology, Hepatology and Endoscopy, University Hospital of Wales, Cardiff, UK; 5 CCMRC, Division of Cancer and Genetics, Henry Wellcome Building, Cardiff University, Cardiff, UK

**Keywords:** T cells, Regulatory T cells, 5T4, Antigen specificity, Cancer

## Abstract

The oncofoetal antigen 5T4 is a promising T cell target in the context of colorectal cancer, as demonstrated by a recent clinical study where 5T4-specific T cell responses, induced by vaccination or cyclophosphamide, were associated with a significantly prolonged survival of patients with metastatic disease. Whilst Th1-type (IFN-γ^+^) responses specific to 5T4, and other oncofoetal antigens, are often readily detectable in early stage CRC patients and healthy donors, their activity is suppressed as the cancer progresses by CD4^+^CD25^hi^Foxp3^+^ regulatory T cells (Treg) which contribute to the immunosuppressive environment conducive to tumour growth. This study mapped the fine specificity of Th1 and Treg cell responses to the 5T4 protein. Surprisingly, both immunogenic peptides and those recognised by Tregs clustered in the same HLA-DR transcending epitope-rich hotspots within the 5T4 protein. Similarly, regions of low Th1-cell immunogenicity also did not contain peptides capable of stimulating Tregs, further supporting the notion that Treg and Th1 cells recognise the same peptides. Understanding the rules which govern the balance of Th1 and Treg cells responding to a given peptide specificity is, therefore, of fundamental importance to designing strategies for manipulating the balance in favour of Th1 cells, and thus the most effective anti-cancer T cell responses.

**Electronic supplementary material:**

The online version of this article (10.1007/s00262-018-2266-1) contains supplementary material, which is available to authorized users.

## Introduction

Despite evidence that progression of human colorectal cancer (CRC) can be controlled by greater infiltration of T cells into the tumour [2], responses to immune-based co-inhibitory receptor (checkpoint) blockade and vaccines in CRC patients harbouring MSI^−^ tumours remain very poor [3]. Vaccination, which depends on the identification of tumour antigens, may prove more effective for the treatment of CRC, particularly in conjunction with immune-modulators. To be effective, a vaccine should induce a robust cytotoxic CD8^+^ T cell (CTLs) response as well as a Th1-type CD4^+^ T cell response. Whilst CTLs can directly recognise and kill tumour cells, provided they express MHC class I molecules, CD4^+^ T cells provide help, but also contribute directly to tumour eradication via direct cytotoxic effects, induction of senescence, or indirect IFN-γ-mediated potentiation of tumouricidal macrophages and inhibition of angiogenesis [4].

Although tumour-specific responses are frequently induced after vaccination, these effects do not often lead to improved clinical outcomes. Interrogations of this limited therapeutic efficacy implicate tumour-induced CD4^+^CD25^hi^Foxp3^+^ T cells (Treg) in suppressing the development of effective anti-tumour immune responses [5–7]. Notably tumour-specific epitopes including LAGE-1_108–120_, recognised by melanoma infiltrating Treg [8], and gp100_369–383_, recognised by circulating Treg [9], have been identified. Therefore, vaccination with tumour-specific peptides may unintentionally inhibit the activation of Th1 cells if tumour antigen-specific Treg are also activated. An NY-ESO-1/ISCOMATRIX™ peptide-based vaccine has been shown to increase pre-existing Treg responses against the NY-ESO-1 tumour antigen resulting in inhibition of CD8^+^ T cell proliferation in vitro [10]. Moreover, Welters and colleagues previously showed that vaccination with HPV-derived long peptides could induce HPV-specific Treg in patients with vulvar and cervical cancers [11, 12].

In this study, we set out to determine whether it is possible to deconstruct the T cell response to a cancer antigen to preserve the ability to stimulate IFN-γ^+^ T cells whilst reducing its capacity to stimulate a Treg response. To do this, we chose the oncofoetal antigen, 5T4, which is a transmembrane glycoprotein expressed in human placenta, absent in other normal tissues, but overexpressed by many adenocarcinomas of the lung, breast and colon [13, 14]. We have previously shown that 5T4-specific T cell responses are observed in around 70% of CRC patients and that these responses decline with disease progression, partly because of Treg activity [15]. In addition, the findings of a recent Phase I/II clinical study performed by our group, indicate that 5T4-specific T cells may confer protection against progression of CRC [16]. 5T4 is, therefore, a relevant candidate antigen for cancer immunotherapy. We explored the immunogenicity of 5T4 with the aim of identifying regions of the protein able to induce IFN-γ^+^ T cells in a diverse population comprising individuals with and without CRC. Furthermore, the ability of Treg populations to modulate these responses and the specificity of their action was investigated.

## Materials and methods

### Patients

Sixty-four patients undergoing colectomy for the removal of a primary colorectal adenocarcinoma were consented before surgery to obtain 30–50 mL peripheral blood samples, collected in 10 mL lithium heparin tubes (BD Biosciences). HLA-type was determined from these samples (Welsh Transplantation and Immunogenetics Laboratory).

### Synthetic peptides

**Fig. 1:**
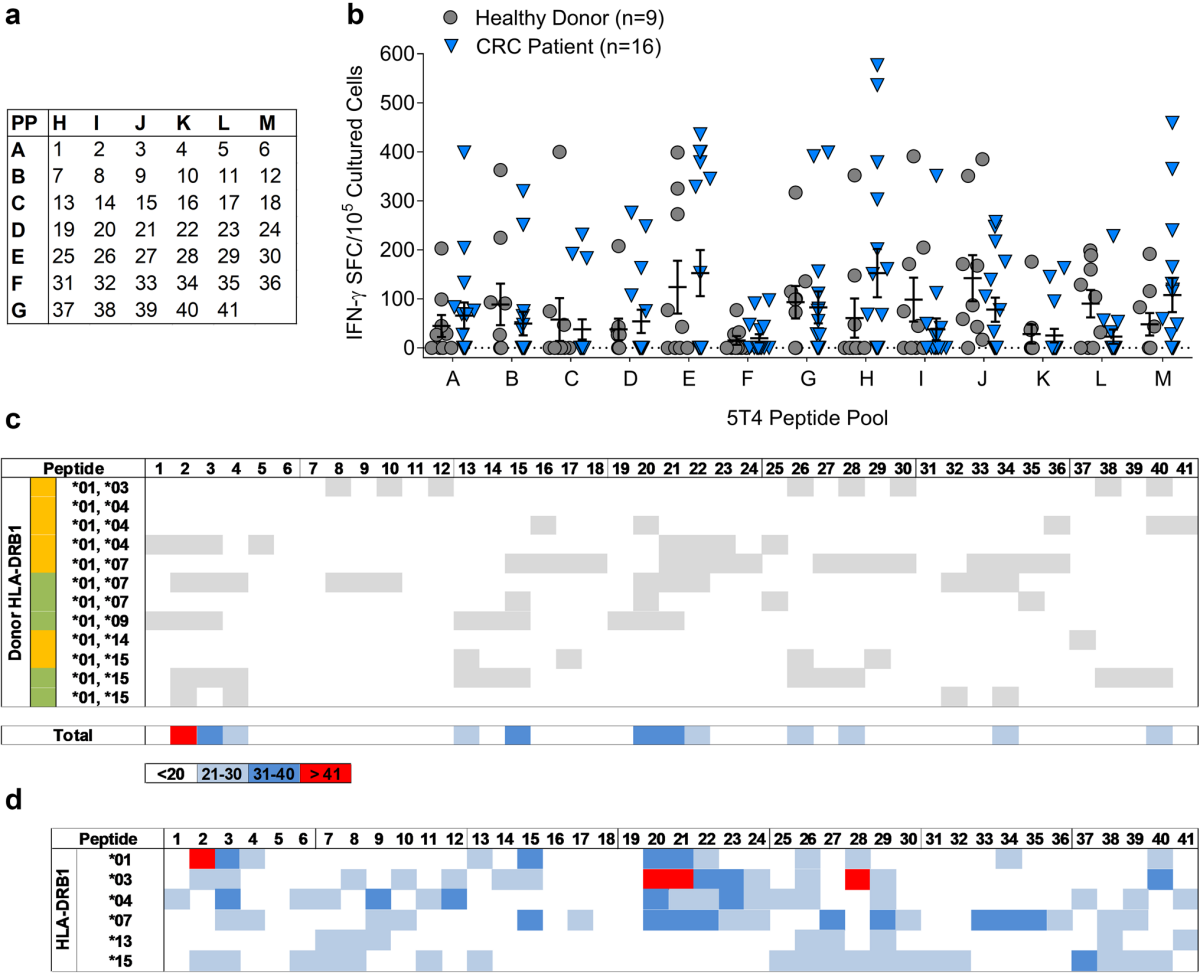
Mapping HLA-DR-restricted 5T4 epitopes. **a** Forty-one 20mer peptides, overlapping by 10 amino acids and spanning the entire 5T4 protein, were incorporated into a peptide pool matrix, whereby each peptide was placed in two pools to aid identification. **b** Following short-term culture of PBMC isolated from healthy donors (*n* = 9) and CRC patients (*n* = 16), 5T4-specific T cell responses were measured by IFN-γ ELISpot, and the number of IFN-γ^+^ spot-forming cells (SFC) per 10^5^ cultured PBMC to each peptide pool collated. **c** HLA-DR heatmap analysis was performed on all patients and healthy donors shown in **b**, in addition to patients and healthy donors used in an earlier study where identical peptide pool experiments were performed [15]. Grey boxes indicate where a putative 5T4-specific response (positive according to pooling matrix analysis) has been identified in that individual, example is shown for all HLA-DRB1*01^+^ donors. Donor status is indicated by the coloured box, green (healthy donors) and orange (cancer patients). **d** This analysis was repeated for all individuals stratified by HLA-DRB1-type. The heatmap illustrates the proportion of individuals putatively responding to each 5T4 peptide

Forty-one 20mer peptides overlapping by 10 amino acids and spanning the entire 5T4 protein were synthesised to > 95% purity (GLBiochem), the sequences of these peptides are shown (Supplementary Table 1). Peptides were dissolved in DMSO at 50 mg/mL and diluted in PBS to 1 mg/mL before use. 13 peptide pools, containing 5–7 peptides, were created in a matrix system where each peptide was present in 2 pools (Fig. 1a). Peptides were added at a final concentration of 5 µg/mL peptide for T cell assays.

### Lymphocyte purification and culture

Peripheral blood mononuclear cells (PBMCs) were isolated by centrifugation of heparinised blood over Lymphoprep (Axis-Shield). Cells were then washed and re-suspended at a concentration of 2 × 10^6^ cells/mL in RPMI (Thermo Fisher) supplemented with 5% batch-tested, pooled human AB serum (Welsh Blood Service), l-glutamine and penicillin/streptomycin. PBMC were plated at 100 µL/well in 96-well plates (Nunc) and cultured in triplicate wells with each 5T4 peptide pool or single 5T4 peptide for 14 days (37 °C, 5% CO_2_). Cells were supplemented with 10 µL CellKine media (Helvetica Healthcare) on day 3 and fresh media containing 20 IU/mL IL-2 on days 7 and 10, as previously described [15]. The re-call antigen tuberculin purified protein derivative (PPD) (Statens Serum Institut) and the mitogen phytohaemagglutinin (PHA) (Sigma–Aldrich) were used as positive controls for T cell activation.

### Depletion of CD25^hi^ regulatory T cells

PBMC were resuspended in 80 µL MACS buffer (PBS, 0.5% bovine serum albumin, 5 mM EDTA) per 10^7^ cells, and 20 µL anti-human CD25 coated beads added (Miltenyi Biotec). Cells were incubated for 15 min at 4 °C, washed and resuspended in 500 µL MACS buffer. MS columns were used for positive selection, both effluent CD25^hi^-depleted and labelled CD25^hi^ cells were collected. The efficiency of depletion was confirmed by flow cytometry. Cells were washed twice before use.

### IFN-γ ELISpot

ELISpots were performed as previously described [15]. Briefly, ELISpot plates (Merck Millipore) were coated overnight with anti-human IFN-γ capture antibody (MabTech). Wells were washed with sterile PBS to remove excess antibody and RPMI containing 5% AB serum was added for 1 h at 37 °C. PBMC lines cultured in triplicate were pooled, washed and plated in the absence or presence of the corresponding 5T4 peptide pool or single peptide. Following 18 h of incubation at 37 °C, 5% CO_2_, cells were removed, plates were washed with PBS and secondary biotinylated anti-human IFN-γ antibody was added. Wells were washed with PBS followed by the addition of streptavidin–alkaline phosphatase (Mabtech). After incubation at room temperature, wells were washed and spots were developed by the addition of a colorimetric substrate kit (Bio-Rad). The reaction was stopped after 15 min, and wells were washed in water. Spots were counted using an automated reader (Autoimmun Diagnostika GMBH, A.I.D.). Counting was checked manually using the ELISpot 5.0 software. The number of spots was normalised to 10^5^ PBMCs. Positive cultured responses were identified as having at least 25 spot-forming cells (SFC) per 10^5^ PBMC, after subtraction of the background, and an increase of at least double the number of spots above background.

### Antibody blocking assays

To determine HLA restriction, IFN-γ secretion was measured in ELISpot assays in the presence or absence of blocking antibodies. 10 µg/mL of anti-HLA-A,B,C (clone w6/32, Biolegend), anti-HLA-DQ (clone 1A3, Leinco) and/or anti-HLA-DR (clone L243, Biolegend) antibodies were added to 5T4-expanded T cells in ELISpot assays. 5T4 peptides were added following 1 h incubation with blocking antibodies.

### Statistics and graphical analysis

GraphPad Prism Version 7 was used for all statistical analyses. Mean values were used for all appropriate results. Unpaired *t* tests were used to analyse quantitative differences between different groups of CRC patients or when comparing healthy donors and CRC patients. FlowJo version 10 was used to analyse flow cytometry data.

## Results

### Detection of pre-existing 5T4-specific CD4^+^ T cells in healthy donors and CRC patients

The peptide specificity of Th1 and Treg cells recognising oncofoetal antigens has not been extensively studied in vitro, possibly due to the scale of experimental assessment necessary to get an accurate picture of responses across multiple donors and HLA alleles. In silico techniques are available, but these ultimately need to be validated in vitro, and simply account for peptide HLA binding, not the potential Th1/Treg make up of responding populations which differs between groups such as cancer patients and healthy donors. Since the goal of this study was to identify regions of the 5T4 protein able to induce IFN-γ^+^ T cells in a diverse population, we first took a high throughput approach using IFN-γ ELISpot assays with a short incubation time, low PBMC number and pools of peptides (as indicated in the matrix shown in Fig. 1a). Responses were measured in healthy individuals and individuals with CRC. Amongst all healthy donors, the strongest overall response was observed against peptide pool J with a mean response of 147 IFN-γ-producing cells/10^5^ cultured PBMC. The weakest overall response was directed against peptide pool F with a mean response of 15 IFN-γ-producing cells/10^5^ cultured PBMC (Fig. 1b). A remarkably similar profile was observed amongst CRC patients, with the highest mean IFN-γ^+^ T cell response again being pool H, and the weakest mean response in pool F. Compared to CRC patients, higher percentages of 5T4-reactive healthy donors were observed for all the peptide pools (Fig. 1b), in line with previous observations from our laboratory examining responses to the 5T4 protein [15].

### Correlating HLA types of patients and healthy donors with peptide-specific T cell responses

Since the number of reported 5T4-derived peptide epitopes is very limited, comprehensive mapping of T cell epitopes was conducted to further assess the usefulness of 5T4 to create peptide-based vaccines. We directed our analysis towards peptides to which reactivity patterns were associated with HLA-DR alleles. Therefore, responses to all putative 5T4 peptides identified from the peptide pool matrix (see example in Supplementary Fig. 2) were mapped according to donor HLA-DRB1 genotype. In addition, this eliminated peptides which may have been overestimated by the pooling matrix methodology, or were presented by HLA molecules other than HLA-DR. After grouping together all patients expressing the same DRB1 allele (example shown for DRB1*01 group in Fig. 1c, remaining HLA types shown in Supplementary Fig. 3), heatmaps revealed that specific sets of putative peptides associate with DRB1*01, *15, *03, *04, *07 and *13 alleles (Fig. 1d). Screening of the entire 5T4 amino acid sequence revealed four immunogenic regions: 5T4_11–40_ (peptides 2–3), 5T4_61–100_ (peptides 7–9), 5T4_191–300_ (peptides 20–29) and 5T4_371–410_ (peptides 38–40). These regions contain overlapping peptides with a reactivity greater than 21% (blue, dark blue or red squares) associated with at least three HLA-DRB1 alleles. In particular, peptides 20 and 21 were highly reactive, being recognised in more than 41% of DRB1*03^+^ donors, as well as 21–40% of DRB1*01, DRB1*04 and DRB1*07 donors. In contrast, amino acid regions 131 to 200 (peptides 14–19) and 301 to 370 (peptides 31–36) contained the fewest epitopes as indicated by the relatively low proportions of individuals responding to peptide pools C and F.

By analysing data across multiple donors using an HLA-DR focused heatmap approach, we were able to identify the broad patterns of reactivity to 5T4. This overcame natural donor to donor differences and variations in reactivity (e.g. between three DRB1*01^+^, *04^+^ donors, Fig. 1c) that likely reflected differences in patient status, age and tumour stage [15]. This analysis emphasised that putative epitopes mediating 5T4 immunogenicity across the population (DRB1*03, DRB1*01, DRB1*04 and DRB1*07 are estimated to cover > 50% European American Population [17]), are not randomly distributed, but are focused in distinct regions of the protein. In the case of self-antigen, such areas have been hypothesised and extensively identified in silico [18–20], but comprehensive in vitro mapping studies of TAAs such as 5T4, are rare, despite their importance to immunotherapy.

### Detailed analysis of individual peptide responses

Following identification of immunogenic regions, peptides were selected for further investigations based on two essential criteria: (i) in vitro putative T cell reactivity in greater than 21% of donors for three HLA-DRB1 groups (from Fig. 1d) and (ii) in silico predicted binding to more than two HLA-DRB1 molecules in NetMHCII 2.2 [21] and IEDB [22] prediction algorithms (Supplementary Fig. 1). Based on this initial criterion, 14 candidate peptides were selected: peptides 2, 3, 15, 20 to 29 and 38. Furthermore, three peptides which showed in vitro reactivity across two HLA-DR groups were added based on the results of NetMHCII 2.2 [21] and IEDB [22] prediction algorithms: Peptides 10 and 12 were predicted to be strong binders to between two and six HLA-DR molecules by either algorithm; p11 was predicted to bind to fewer HLA-DR molecules but was included as it overlapped with both peptides (Supplementary Fig. 1). In total, 17 peptides were taken forward for further analysis.

**Fig. 2:**
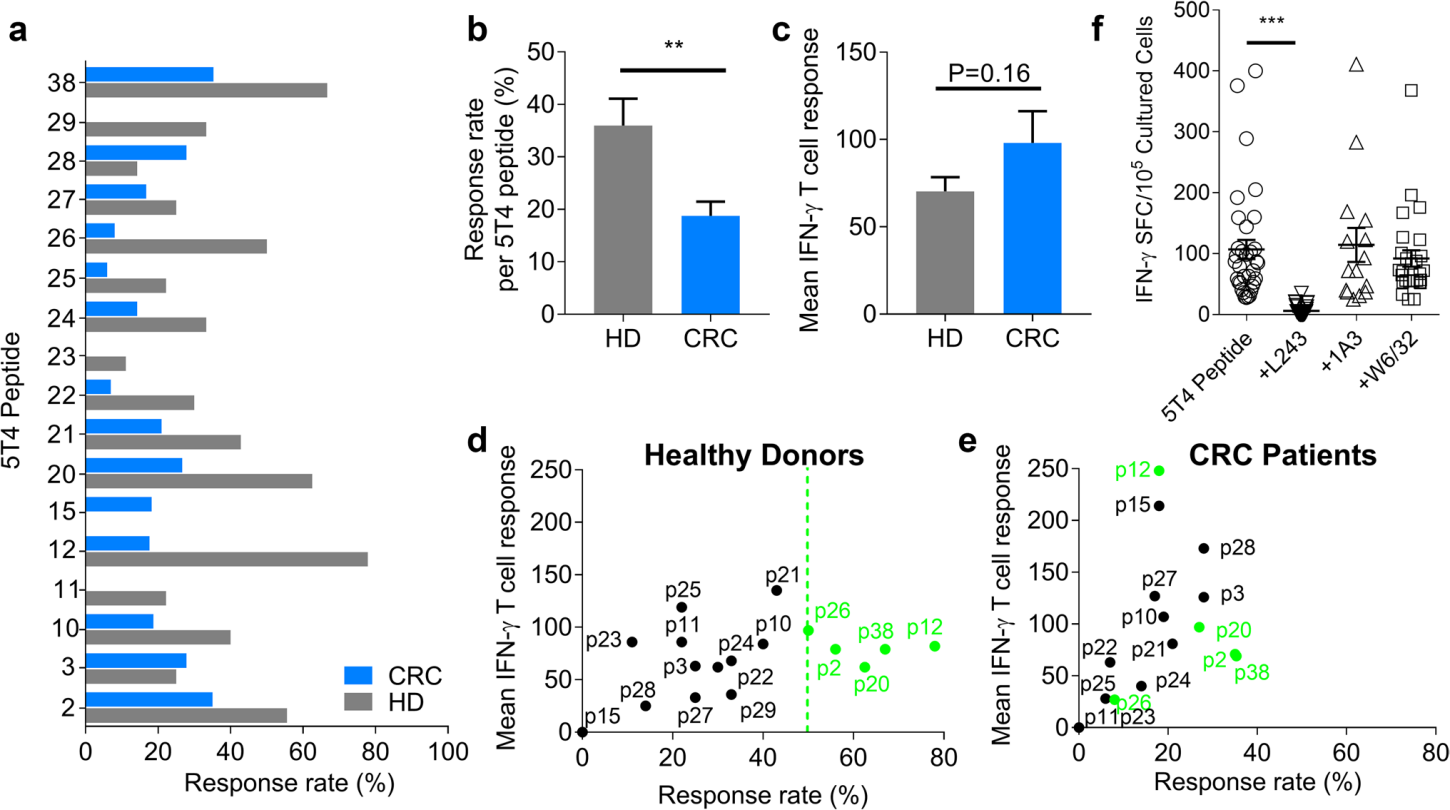
Characterising peptide-specific 5T4 T cell responses generated by healthy donors and CRC patients. The percentage of healthy donors and CRC patients who had positive responses to each candidate 5T4 peptide (**a**) and the response rate to all peptides (**b**). **c** The mean IFN-γ^+^ T cell response to each candidate peptide, defined as the number of SFC/10^5^ cultured PBMC, amongst healthy donors and CRC patients. The mean T cell response and response rate (%) amongst healthy donors (**d**) and CRC patients (**e**) is graphed together. Peptides which are recognised with high frequency (> 50%) in healthy donors are highlighted in green. **f** PBMC generating 5T4 peptide specific responses amongst all donors were re-stimulated with the 17 immunogenic 5T4 peptides in the presence of MHC blocking antibodies (L243, 1A3 and W6/32 which block HLA-DR, HLA-DQ and HLA-A/B/C respectively). Significant differences are indicated (***P < 0.0001). Results are expressed as the number of IFN-γ^+^ SFC/10^5^ cultured PBMC after subtracting background spots

In healthy donors, all peptides except for p15 induced IFN-γ production, the most commonly recognised being p12 (78%), p38 (67%), p20 (63%), p2 (56%) and p26 (50%) (Fig. 2a). In CRC patients, all peptides other than p11 and p29 induced a response, the most commonly detected being p38 (35%), p2 (33%), p3 (28%) and p28 (28%) (Fig. 2a). Peptides 10 and 12 were more immunogenic than p11, as predicted by the algorithms. For all peptides tested, the percentage of responders was significantly higher in healthy donors than in CRC patients (HD 36.0% ± 5.1 vs. CRC 18.8% ± 2.7, *P* = 0.0079; Fig. 2b). This is consistent with early observations using peptide pools (Fig. 1).

The mean IFN-γ^+^ T cell response generated to each candidate 5T4 peptide by all donors was also calculated. Although there was a trend for CRC patients to mount higher IFN-γ^+^ responses over healthy donor controls, this was not significant (HD 70.4 SFC ± 8.1 vs. CRC 98.1 SFC ± 18.2, *P* = 0.16; Fig. 2c). Therefore, whilst the overall response rate to 5T4 peptides was lower in CRC patients, the mean T cell response mounted tended to be higher. Based on the magnitude of positive responses and response rates (Fig. 2d, e), peptides 2, 12, 20, 26 and 38 emerge as immunodominant 5T4 peptides in healthy controls (≥ 50% of donors, shown by the green dotted line in Fig. 2d). All five peptides are also recognised by CRC patients but to a noticeably lesser extent (5–38%). The same trend was observed for the other peptides, except for p3 and p28, where responses were always less frequent or lower in magnitude for CRC patients (further details of immunogenic peptides in Supplementary Table 2).

To assess the extent of HLA-restriction in responses to our group of candidate peptides, we performed HLA blocking assays in both CRC patients and healthy donors (Fig. 2f). There was at least a 50% reduction in IFN-γ^+^ T cell response when testing individual peptides in the presence of anti-HLA-DR blocking antibody (L243) (Fig. 2f). In contrast, addition of anti-HLA-DQ (1A3) or anti-HLA-A/B/C (W6/32) did not affect peptide responses generated in any instance. This result validated our allele focused screening approach, and confirmed that most responses were mediated by HLA-DR.

### 5T4-specific Treg epitopes

**Fig. 3:**
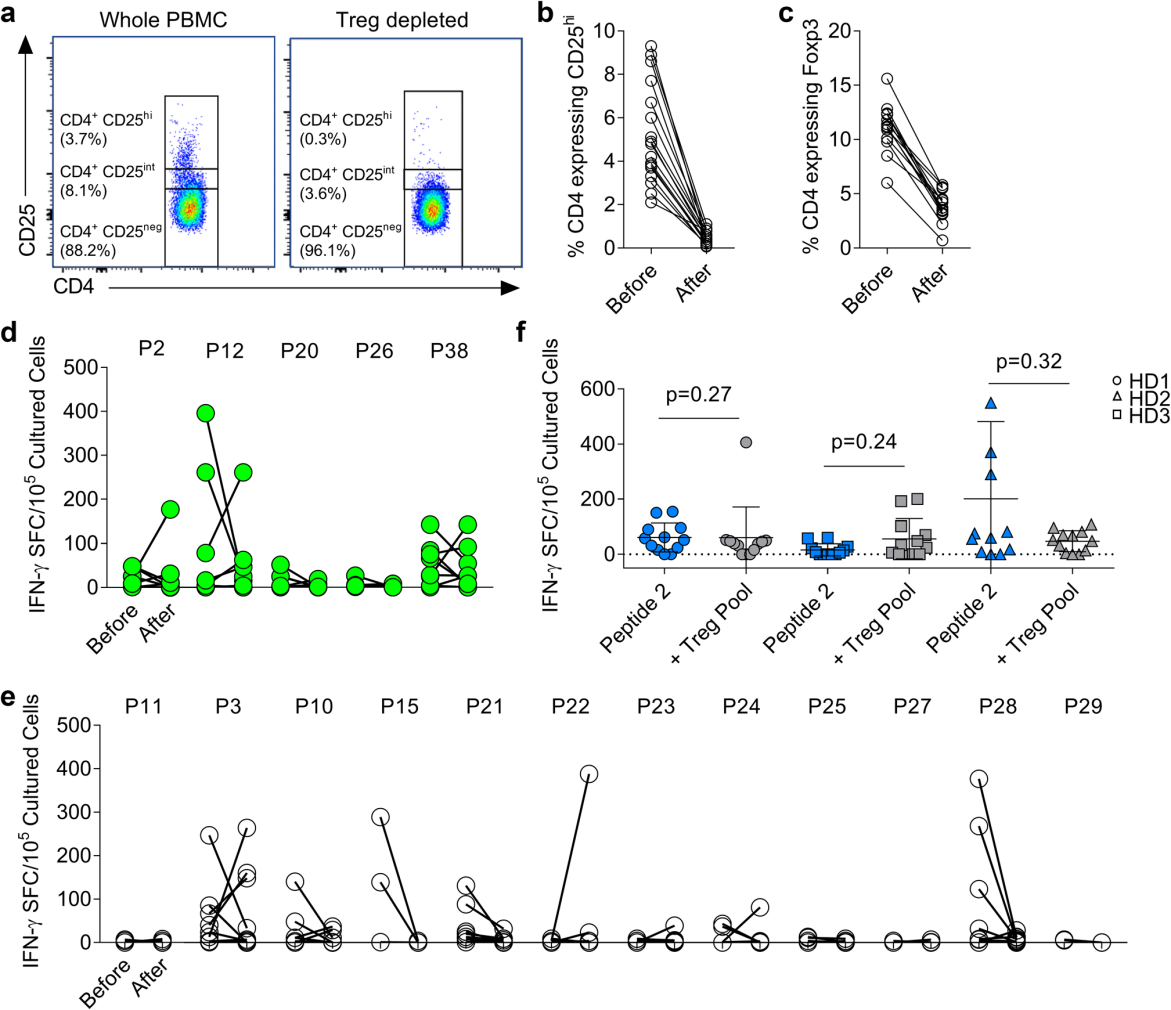
Regulatory T cells do not suppress all effector anti-5T4 T cell responses. **a** Whole PBMC were depleted of CD25^hi^ T cells, a representative example of the FACS plots confirming adequate depletion is shown. The proportion of CD4^+^ T cells expressing CD25^hi^ (**b**) or Foxp3 (**c**) in all donors is shown before and after depletion. PBMC and Treg-depleted PBMC from CRC patients were stimulated with each of the five immunogenic peptides (**d**) and 12 candidate peptides (**e**). The IFN-γ spot-forming cells/10^5^ cultured PBMC, after subtraction of background spots, was measured before and after the depletion of CD25^hi^ T cells. **f** PBMC were cultured with 5T4 peptide 2, with or without the ‘Treg peptide pool’ before restimulation with peptide 2 alone on IFN-γ ELISpot. Assay was carried out in three healthy donors (twelve PBMC lines per condition)

We have previously demonstrated that CRC patients have higher numbers of Treg with a suppressive phenotype relative to healthy individuals [7, 15, 23] and that anti-5T4 responses are more likely to be controlled by these cells in CRC patients. Here, we set out to determine whether peptides stimulating an IFN-γ T cell response also stimulated Treg. To do this, CD25^hi^ cells were depleted from PBMCs using Miltenyi MACS beads and T cell responses to each peptide were compared in depleted and non-depleted cultures. As shown in Fig. 3a–c, this method of depletion always significantly decreased the proportion of CD25^hi^ cells (before: 5.20% ± 1.18 vs. after: 0.49% ± 0.16, Fig. 3a, b) corresponding with a decrease of Foxp3^+^ cells (before: 11.16% ± 1.28 vs. after: 3.79% ± 0.80, Fig. 3c).

In CRC patients, responses to the 17 candidate peptides were assessed in cultured IFN-γ^+^ ELISpot assays pulsed with single peptides, in the presence or absence of CD25^hi^ T cells. With respect to the immunodominant peptides 2, 12, 20, 26 and 38, new or increased responses were observed against peptides 2, 12 and 38 in at least one donor (Fig. 3d) indicating that at least in some donors, the same peptides could induce both Treg activity and IFN-γ producing T cells. Other instances of Treg suppression were observed for peptides 3, 22, 23 and 24. Finally, responses to p28, which were observed largely in CRC patients (Fig. 2d, e), were reduced by Treg-depletion (Fig. 3e). Where existing responses were abrogated by Treg depletion (also occurred for peptides 3, 10, 15, and 24, in some donors), this appeared to be independent of donor HLA-type (Supplementary Table 3). The most likely explanation was a depletion of activated effector cells with high CD25 expression.

Overall, Treg-mediated control of 5T4-specific responses was observed in 8/14 (57.1%) CRC patients tested. These data provide evidence for Treg-mediated inhibition of Th1 cells of the same peptide specificity but also indicate that some but not all peptides induce Treg responses. Whether the same 9mer epitopes within each 20mer were the focus of Th1 and Treg immunogenicity cannot be conclusively determined from the experimental data. However, for the immunodominant peptides (2, 26 and 38) the same core 9mer was predicted to bind with highest affinity to all HLA-DR alleles, suggesting within certain peptides a single epitope may dominate HLA-DR mediated immunogenicity (Supplementary Table 4).

Our reductive approach indicates that Treg can suppress in an antigen-specific manner and likely share peptide specificity with conventional T cells. However, it is also possible that Treg may be stimulated by a given peptide but suppress in an antigen non-specific manner. An experiment was, therefore, designed to determine whether those 5T4 peptides that rarely stimulate IFN-γ production (as indicated in Fig. 1d), might in fact represent Treg epitopes. Peptides 5, 16, 18 and 19, which rarely induced IFN-γ responses (< 20% of donors), were selected and added together to make a putative ‘5T4 Treg pool’. As strong responses to the control antigen PPD were not impacted by addition of this Treg pool (Supplementary Fig. 4), we hypothesised that the suppressive effect of the 5T4 Treg pool may only impact responses to other 5T4 peptides. Cultured responses to peptide 2, to which most healthy donors respond (predicted to bind HLA-DRB1*01), were used to design an in vitro suppression assay.

PBMC from three healthy DRB1*01^+^ donors were expanded to peptide 2 (twelve replicates per donor) in the presence or absence of the putative Treg pool (Fig. 3f). Although some donor variation in response magnitudes were observed, no significant differences in the number of positive lines, or magnitude of responses in the presence of the putative Treg pool were observed. Thus, we hypothesised that these peptides were not stimulators of epitope-specific Treg, but simply poorly immunogenic relative to the rest of the 5T4 protein.

## Discussion

It has become increasingly clear that the oncofoetal antigen 5T4 is a promising T cell target in the context of CRC. In a recent clinical study, patients with inoperable CRC received either cyclophosphamide alone to deplete Treg, a vaccine comprising 5T4-expressing modified vaccinia Ankara (TroVax) or a combination of both [16]. Both treatments were shown to independently induce 5T4-specific responses which were associated with prolonged patient survival. We set out to deconstruct these highly beneficial responses further, first by identification of specific peptides responsible for IFN-γ^+^ immunogenicity, and second by evaluating the extent and specificity of Treg mediated suppression. Such knowledge could be utilised to improve therapeutic strategies built on responses to TAAs by altering the balance in activity of Th1 and Treg populations.

Using a panel of overlapping peptides covering the 5T4 protein and blood samples from a cohort of CRC patients and healthy controls, we found 5 immunogenic regions within the 5T4 protein and 17 peptides recognised by T cells from healthy controls and, to a lesser extent, CRC patients. Further analysis revealed a hierarchy of peptides recognised by healthy donors and CRC patients, with p2, p20 and p38 consistently immunogenic in both groups. These peptides, together with p3, p26 and p28, to which responses were also often observed in CRC patients, form a useful group of peptides for tracking 5T4-specific T cells responses during disease progression and in response to vaccination. Only a few TAAs with high immunogenicity across the population have been reported previously; NYESO-1 [10], MELOE-1 [24–26] and Survivin [9]. Recently, the discovery and characterisation of novel TAAs has been superseded by a strong focus on the discovery and characterisation of neoepitopes, which although efficacious at the level of the individual, are not useful for universal cancer vaccines applicable to the population.

The pan-HLA-DR nature of the immunogenic regions detailed here confirms the suitability of 5T4 for cancer vaccination. The widespread in vitro immunogenicity of key regions may arise from several competing factors: first, the long length of the 5T4 peptides used (20 amino acids) make it possible that several epitopes of different 9-mer core sequences (with distinct HLA-DR restriction) are present within these peptides enabling many donors of different HLA types to respond. Second, the peptides identified may be promiscuous and thus able to bind various HLA-alleles. It has previously been shown that a short epitope (14-mer) derived from HCA587 tumour Ag can stimulate T cells in the context of more than one HLA class II allele [27], while for viral antigens such as influenza haemagglutinin, the most immunogenic HLA class II epitope exhibits strong binding to nearly all HLA-DR alleles [28]. Thirdly, recognition of these peptides may have arisen by T cell cross-reactivity with common microbial epitopes [29]. The most immunogenic peptides may exhibit molecular mimicry to highly immunogenic microbial peptides when presented in the context of specific HLA alleles. Such microbial epitopes could have primed large Th1 memory pools during infection, only a fraction of which would need 5T4 cross-reactive TCRs to elicit the immunogenicity described here.

While T cell responses to 5T4 were readily observed in CRC patients, these were diminished compared to those observed in healthy controls. It is clear, therefore, that mechanisms of peripheral tolerance do not prevent induction of 5T4-specific T cell responses but that these responses are suppressed due to the presence of the cancer. Several cancer-associated immunosuppressive mechanisms have been described including expression of co-inhibitory receptors by effector T cells, suppression by Treg, myeloid-derived suppressor cells and/or immunosuppressive cytokines. It is also possible that CRC patients have a more limited TCR repertoire of 5T4-specific T cells, as has been shown for the TRAG-3 tumour Ag in melanoma and breast cancer patients [30].

This study focused on Treg-mediated immunosuppression since we have previously observed that Treg do suppress 5T4-specific T cell responses in CRC patients and that this is more likely to be observed during the later stages of disease. Examining T cell responses to individual peptides as described herein enabled us to examine the peptide specificity of Treg in CRC patients. Our results revealed that Treg and Th1 cells share the same specificity for some, but not all 5T4 peptides. Furthermore, whether an individual peptide is recognised by Treg and/or Th1 cells varies between patients, rendering the possibility of creating a universal 5T4 vaccine including beneficial T helper peptides and excluding detrimental Treg epitopes of the same specificity very challenging. Some studies have reported that whilst Treg are antigen-specific, their suppressive activities are antigen non-specific. For instance, upon activation, HPV-specific Treg can suppress influenza specific-Th1 cell activity [31]. Whilst a previous study by Bonertz and colleagues showed that Treg can recognise several tumour Ags, however, their ability to suppress in an Ag non-specific manner was not tested [32]. As well as examining the capacity of Tregs to be activated in response to peptides recognised by Th1 cells, we tested whether regions of the 5T4 protein, which consistently failed to activate IFN-γ^+^ T cells, contain peptides recognised by Treg. These assays did not show non-immunogenic regions to harbour Treg epitopes, but instead reiterated that Th1 and Treg populations must share some specificity and the balance between populations likely determines the magnitude of the overall response [33].

Overall, this study has revealed numerous peptides, which may be recognised by both Th1 and Treg cells. These data are reminiscent of those published previously by Welters et al. who showed that vaccination with HPV-derived long peptides induce HPV-specific Th1 and Treg responses in patients with different type of cancers [11, 12] where a high Th1 to Treg ratio was predictive of a good clinical response. In this case, a combination of vaccine and an agent to downmodulate Treg might be required. Therapeutic agents that may selectively target CD25^hi^ populations including Treg, such as cyclophosphamide, are attractive for this purpose. Studies of chronic infection in mice have shown that the context of antigen exposure alters the balance between Th1 and Treg cells of a given specificity [33]. A better understanding of the rules governing this balance should prove useful for informing strategies designed to minimise induction of tumour antigen-specific Treg cells.

## Electronic supplementary material

Below is the link to the electronic supplementary material.

Supplementary material 1 (PDF 492 KB)

## Abbreviations

Ag: Antigen
CRC: Colorectal cancer
CTL: Cytotoxic lymphocyte
ELISpot: Enzyme-linked immunospot
FACS: Fluorescence-activated cell sorting
Fluorospot: Fluorescent immunospot
Foxp3: Forkhead box p3
HD: Healthy donor
HLA: Human leukocyte antigen
IEDB: Immune epitope database
IFN-γ: Interferon-γ
MHC: Major histocompatibility complex
PBMC: Peripheral blood mononuclear cell
PPD: Purified protein derivative
SEM: Standard error of the mean
SFC: Spot-forming cell
TAA: Tumour-associated antigen
Th1: T helper-1 cell
Treg: Regulatory T cell
