# Supplementary material for: The nature of the human T cell response to the cancer antigen 5T4 is determined by the balance of regulatory and inflammatory T cells of the same antigen-specificity: implications for vaccine design
Source: Cancer Immunol Immunother. 2018 Nov 7;68(2):247–56. doi: 10.1007/s00262-018-2266-1 (PMC6394487; doi:10.1007/s00262-018-2266-1)
Supplement: Supplementary file 1 — Supplementary material 1 (PDF 492 KB) [file 262_2018_2266_MOESM1_ESM.pdf]

No.	Sequence	No.	Sequence	No.	Sequence
1	MPGGCSRGPAAGDGRLRLAR	15	LPSLRQLDLSHNPLADLSPF	29	LQGLPHIRVFLDNNPWVCDC
2	AGDGRLRLARLALVLLGWVS	16	HNPLADLSPFAFSGSNASVS	30	LDNNPWVCDCHMADMVTWLK
3	LALVLLGWVSSSSPTSSASS	17	AFSGSNASVSAPSPLVELIL	31	HMADMVTWLKETEVVQGKDR
4	SSSPTSSASSFSSSAPFLAS	18	APSPLVELILNHIVPPEDER	32	ETEVVQGKDRLTCAYPEKMR
5	FSSSAPFLASAVSAQPPLPD	19	NHIVPPEDERQNRSFEGMVV	33	LTCAYPEKMRNRVLLELNSA
6	AVSAQPPLPDQCPALCECSE	20	QNRSFEGMVVAALLAGRALQ	34	NRVLLELNSADLDCDPILPP
7	QCPALCECSEAARTVKCVNR	21	AALLAGRALQGLRRLELASN	35	DLDCDPILPPSLQTSYVFLG
8	AARTVKCVNRNLTEVPTDLP	22	GLRRLELASNHFLYLPRDVL	36	SLQTSYVFLGIVLALIGAIF
9	NLTEVPTDLPAYVRNLFLTG	23	HFLYLPRDVLAQLPSLRHLD	37	IVLALIGAIFLLVLYLNRKG
10	AYVRNLFLTGNQLAVLPAGA	24	AQLPSLRHLDLSNNSLVSLT	38	LLVLYLNRKGIKKWMHNIRD
11	NQLAVLPAGAFARRPPLAEL	25	LSNNSLVSLTYVSFRNLTHL	39	IKKWMHNIRDACRDHMEGYH
12	FARRPPLAELAALNLSGSRL	26	YVSFRNLTHLESLHLEDNAL	40	ACRDHMEGYHYRYEINADPR
13	AALNLSGSRLDEVRAGAFEH	27	ESLHLEDNALKVLHNGTLAE	41	YRYEINADPRLTNLSSNSDV
14	DEVRAGAFEHLPSLRQLDLS	28	KVLHNGTLAELQGLPHIRVF		

Supplementary Table 1 5T4 peptide sequences. Forty-one 20mer peptides, overlapping by 10 amino acids, were synthesised to >95% purity to span the entire 5T4 sequence

	Immunogenicity		Origin	Sequence	Domain origin
p2	HD	CRC	$5T4_{11-30}$	AGDGRLRLARLALVLLGWVS	Signal peptide
p3		CRC	$5T4_{21-40}$	LALVLLGWVSSSSPTSSASS	Signal peptide/Extracellular
p12	HD		$5T4_{111-130}$	FARRPPLAELAALNLSGSRL	Extracellular LRR 1-2
p20	HD	CRC	$5T4_{191-210}$	QNRSFEGMVVAALLAGRALQ	Extracellular LRR 4-5
p26	HD		$5T4_{251-270}$	YVSFRNLTHLESLHLEDNAL	Extracellular LRR 6-7
p28		CRC	$5T4_{271-290}$	KVLHNGTLAELQGLPHIRVF	Extracellular LRR 7-LRRCT
p38	HD	CRC	$5T4_{371-390}$	LLVLYLNRKGIKKWMHNIRD	Transmembrane/Intracellular

Supplementary Table 2 Further details of peptides identified as immunogenic from the 5T4 protein

CRC Patients	HLA-DRB1	Peptide 3 (+)	Peptide 3 (-)	Peptide 10 (+)	Peptide 10 (-)	Peptide 15 (+)	Peptide 15 (-)	Peptide 24 (+)	Peptide 24 (-)	Peptide 28 (+)	Peptide 28 (-)
	**DR1+**										
**MB30**	***01, *13**	0	0	0	0	139	*0*	0	0	0	**28**
**MB51**	***01, *03**	1	0	11	0	0	0	0	0	0	0
**MB65**	***01, -**	40	**160**	48	*0*			1	**81**	1	1
	**DR15+**										
**MB26**	***15, *04**	24	4			289	*4*			123	*8*
**MB64**	***15, -**	3	7	3	0			1	3	32	*1*
**MB66**	***04. *15**	67	**148**	140	*11*			37	*1*	8	13
**MB49**	***03, *15**	85	*0*	0	0	0	0	0	0	0	0
	**DR3+**										
**MB29**	***03, *04**	247	*33*	11	**37**			43	*0*	268	*5*
**MB41**	***03, *04**	0	0	0	0	0	0	0	0	0	0
**MB48**	***03, *13**	0	0	0	0	0		0	0	0	0
**MB49**	***03, *15**	85	*0*	0	0	0	0	0	0	0	0
**MB51**	***01, *03**	1	0	11	0	0	0	0	0	0	0
**MB62**	***03, -**	3	1	0	**29**					5	3
	**DR4+**										
**MB26**	***15, *04**	24	4			289	*4*			123	*8*
**MB28**	***04, *07**	1	0	3	1			0	0	7	0
**MB46**	***04, *13**	0	0	0	0	0	0	0	0	0	0
**MB66**	***04. *15**	67	**148**	140	*11*			37	*1*	8	13
**MB29**	***03, *04**	247	*33*	11	**37**			43	*0*	268	*5*
**MB41**	***03, *04**	0	0	0	0	0	0	0	0	0	0
	**DR13+**										
**MB30**	***01, *13**	0	0	0	0	139	0	0	0	0	**28**
**MB48**	***03, *13**	0	0	0	0	0		0	0	0	0
**MB46**	***04, *13**	0	0	0	0	0	0	0	0	0	0
	**Other DR**										
**MB27**	***08, *14**	13	**264**			1	0			377	*12*

(Column headings as printed: **Peptides** 3, 10, 15, 24, 28; **Treg replete (+) or following Depletion (-)**.)

Supplementary Table 3 Response changes upon Treg depletion, stratified by donor HLA-type. Peptides are listed at the top. The numbers represent the spot-forming cells /10^5 cultured PBMC after subtraction of background spots, in the presence (+ sign) or absence (- sign) of $CD25^{hi}$ cells. Underlined numbers indicate positive responses. Blue numbers indicate an increase of at least 50% in depleted cells, red new responses and *italic* a decrease, compared to replete cells

		HLA-DRB						
Strongest Predicted Core 9-mer		*0101	*0301	*0401	*0701	*1302	*1501	20mer
	Peptide-2	**LRLARLALV**	**LRLARLALV**	**LRLARLALV**	**LRLARLALV**	**LRLARLALV**	**LRLARLALV**	AGDGR**LRLARLALV**LLGWVS
	Affinity (nM)	*57*	*1162*	*771*	*182*	*154*	*158*	
	Peptide-12	**LAELAALNL**	AALNLSGSR	LAALNLSGS	**LAELAALNL**	AALNLSGSR	**LAELAALNL**	FARRPP**LAELAALNL**SGSRL
	Affinity (nM)	*175*	*5508*	*1338*	*807*	*1114*	*765*	
	Peptide-20	**VAALLAGRA**	VVAALLAGR	FEGMVVAAL	FEGMVVAAL	**VAALLAGRA**	**VAALLAGRA**	QNRSFEGMV**VAALLAGRA**LQ
	Affinity (nM)	*60*	*3901*	*914*	*356*	*1475*	*264*	
	Peptide-26	**LTHLESLHL**	**LTHLESLHL**	**LTHLESLHL**	**LTHLESLHL**	**LTHLESLHL**	**LTHLESLHL**	YVSFRN**LTHLESLHL**EDNAL
	Affinity (nM)	*68*	*3774*	*381*	*214*	*827*	*129*	
	Peptide-38	**LVLYLNRKG**	**LYLNRKGIK**	**LVLYLNRKG**	**LVLYLNRKG**	**LYLNRKGIK**	**LVLYLNRKG**	LLV**LYLNRKGIK**KWMHNIRD
	Affinity (nM)	*421*	*944*	*1837*	*1183*	*431*	*119*	

Supplementary Table 4 Prediction of the strongest binding core from the five most immunogenic 20mer peptides (2, 12, 20, 26 and 38) using the NetMHCII pan 3.2 prediction tool (updated 2018, reference [1]). The strongest predicted core and corresponding affinity prediction in nanomolar (nM) are shown for each 20mer peptide and HLA-DRB1 allele. The dominant core from each 20mer across all alleles is in bold text and underlined within the parent peptide in the far-right column

HLA-DRB1	Representation	
	**Cancer Patients**	**Healthy Donors**
***01**	24%	33%
***03**	17%	27%
***04**	48%	27%
***07**	21%	47%
***13**	21%	7%
***15**	34%	33%

Supplementary Table 5 HLA-DRB1 allele representation across patients and healthy donors used to create DR specific heatmaps

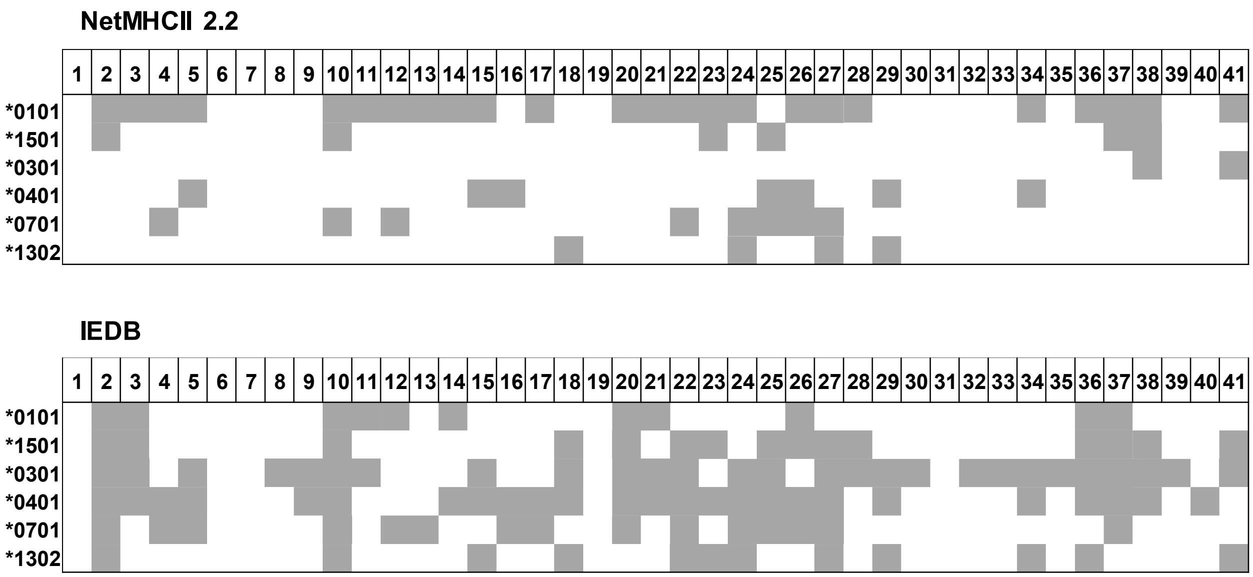

Algorithm	Peptides binding to 3 HLA-DR alleles	Peptides matching to heat map	Peptides not Matching to heat map	Peptides missed
**NetMHC2.2**	6	5	1	14
**IEDB**	20	13	7	6

Supplementary Fig.1 *In silico* predicted MHC class II-restricted peptides of 5T4 protein. Grey boxes indicate predicted strong binders and white boxes non-binders. Default prediction thresholds were used for both algorithms to predict strong binders; IC50 <500 nM for NetMHCII 2.2 [2], percentile rank below 10% for IEDB [3]

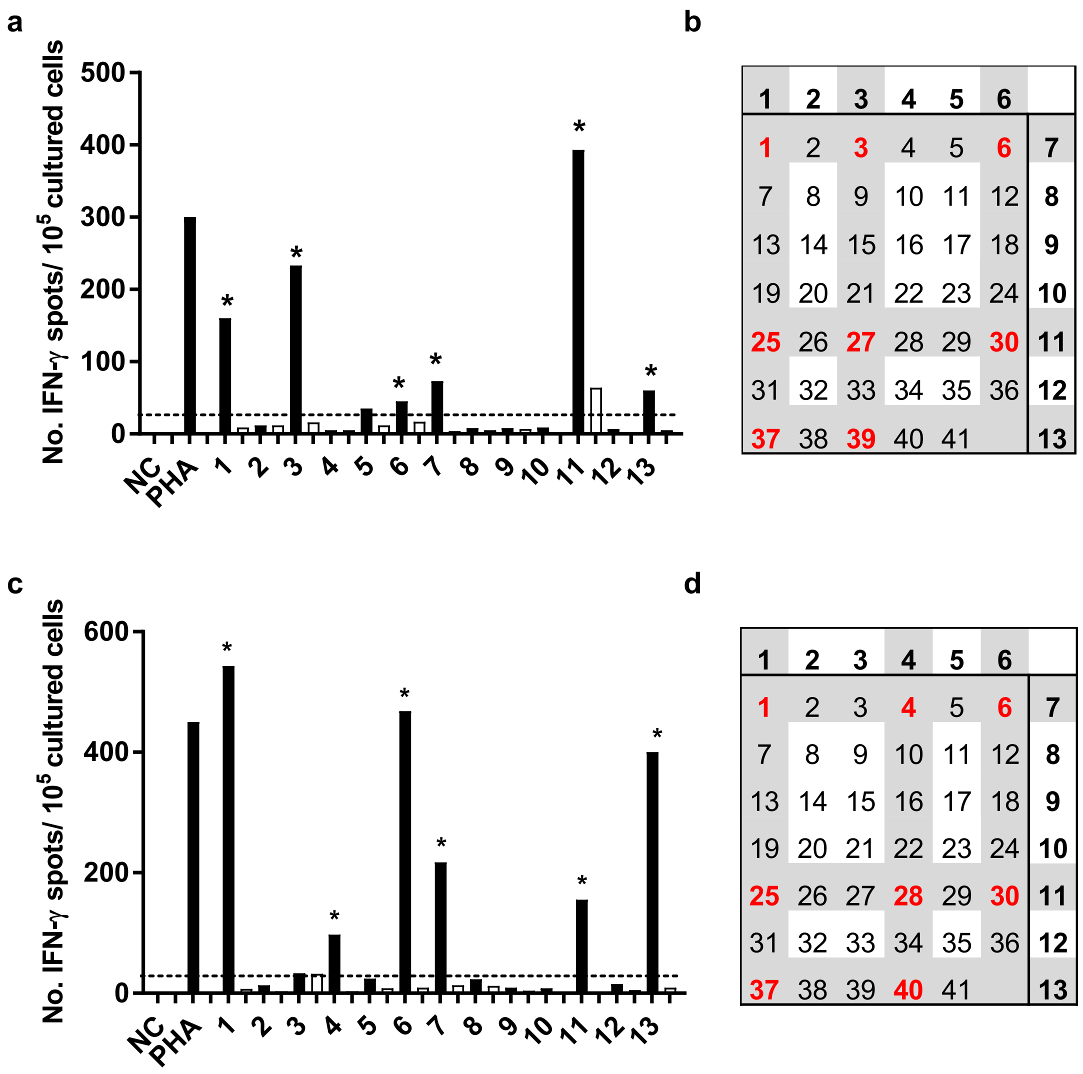

Supplementary Fig.2 Determining candidate 5T4 peptides using a peptide matrix. After 12 days of stimulation, specific responses were measured in an IFN-γ ELISpot assay, data shown for two patients (**a**) and (**c**). The matrix allows for the rapid identification of putative peptides (highlighted in red). Two representative examples are given with CRC patients (**a**), (**b**) MB3 and (**c**), (**d**) MB12

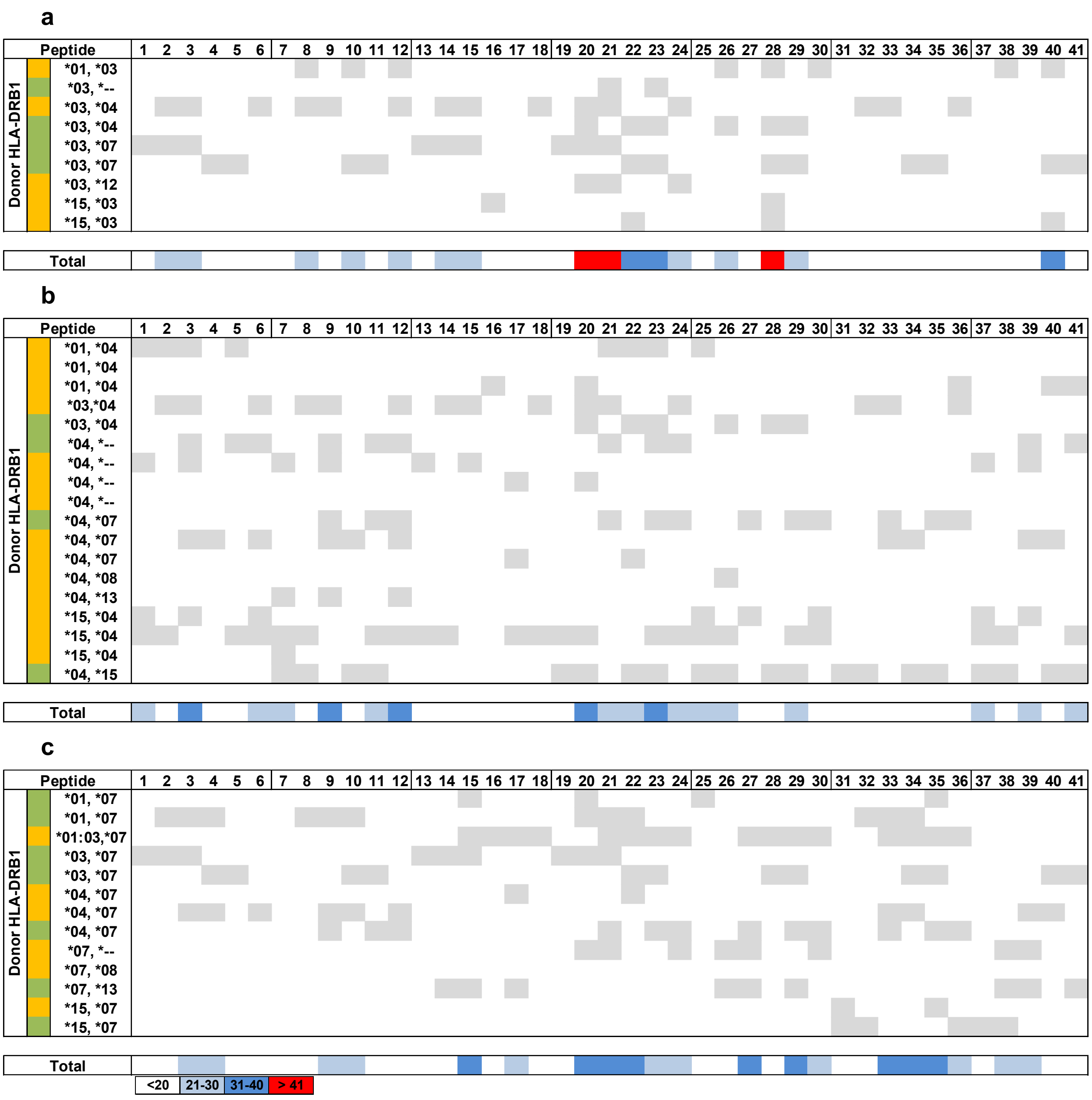

Supplementary Fig.3

d

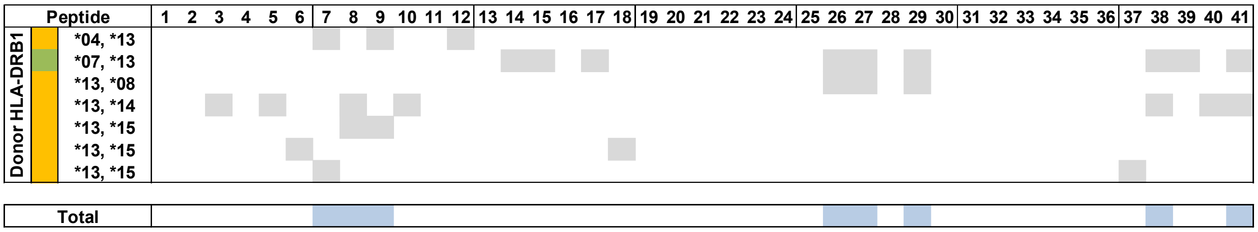

e

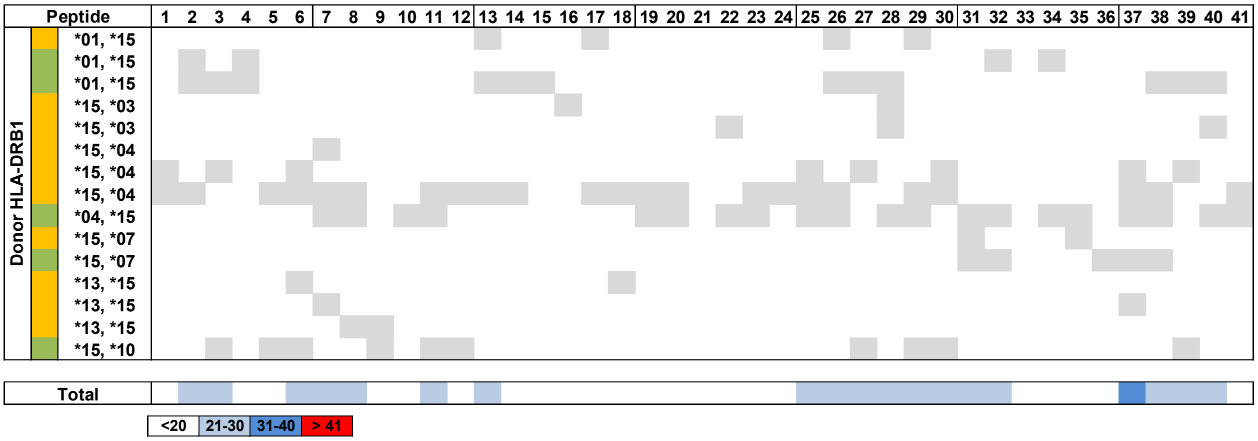

Supplementary Fig.3 Heatmaps of responses stratified by each HLA-DRB1 group. HLA-DRB1*01 was shown in Fig.1d. Grouped by HLA-DRB1 alleles: (**a**) HLA-DRB1*03, (**b**) HLA-DRB1*04, (**c**) HLA-DRB1*07, (**d**) HLA-DRB1*13, (**e**) HLA-DRB1*15. Donor status is indicated by the orange or green box on the far left of each heatmap. Healthy in green and cancer in orange

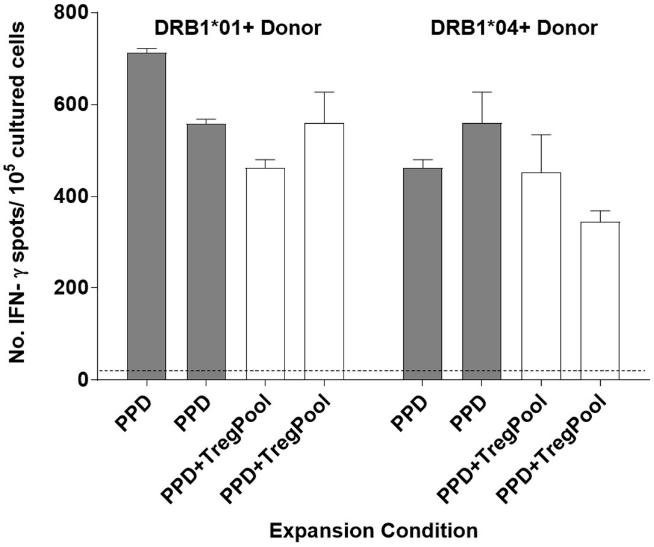

Supplementary Fig.4 PPD responses in the presence or absence of the Treg pool. Two healthy donors, one HLA-DRB1*01^{+} and HLA-DRB1*04^{+} are shown. Four separate PBMC lines per donor were raised to the control antigen PPD, two in the presence of a Treg pool (PPD+TregPool). Lines were re-stimulated with PPD in duplicate on IFN-γ ELISpot, and background subtracted as previously shown

Supplementary references

1. Jensen KK, Andreatta M, Marcatili P, et al (2018) Improved methods for predicting peptide binding affinity to MHC class II molecules. Immunology 154:394–406 . doi: 10.1111/imm.12889
2. Nielsen M, Lund O (2009) NN-align. An artificial neural network-based alignment algorithm for MHC class II peptide binding prediction. BMC Bioinformatics 10:296 . doi: 10.1186/1471-2105-10-296
3. Vita R, Overton JA, Greenbaum JA, et al (2015) The immune epitope database (IEDB) 3.0. Nucleic Acids Res 43:D405–D412 . doi: 10.1093/nar/gku938
